# Supplementary material for: The Drosophila F-box protein Fbxl7 binds to the protocadherin Fat and regulates Dachs localization and Hippo signaling
Source: eLife. 2014 Aug 8;3:e03383. doi: 10.7554/eLife.03383 (PMC4144329; doi:10.7554/eLife.03383)
Supplement: Supplementary file 1. — Localization of FLAG-Fbxl7 and vesicle markers. List of tagged proteins that localize to vesicles, are related to vesicle trafficking, and proteins interacting with Fat. Colocalization was assessed with FLAG-Fbxl7 in the wing imaginal disc. DOI: http://dx.doi.org/10.7554/eLife.03383.019 [file elife03383s001.pdf]

Supplementary File 1 - Localization of FLAG-Fbxl7 and vesicle markers. List of tagged proteins that localize to vesicles, are related to vesicle trafficking, and proteins interacting with Fat. Colocalization was assessed with FLAG-Fbxl7 in the wing imaginal disc. BL indicates Bloomington Drosophila Stock Center.

Gene	Localization	Overlap w/ Fbxl7?	BL #	Stock genotype	Ref
Rab1	Endoplasmic reticulum	no	24104	P{UAST-YFP.Rab1}Mes2[01]	(Zhang et al., 2007)
Rab2		no	23246	P{UAST-YFP.Rab2}l(3)neo38[02]	(Zhang et al., 2007)
Rab3		no	9763	P{UASp-YFP.Rab3}4EHP[05b]	(Zhang et al., 2007)
Rab4	Early endosomes	no	9767	P{UASp-YFP.Rab4}Sap-r[32]	(Zhang et al., 2007)
Rab5	Early endosomes	no	24616	P{UASp-YFP.Rab5}02	(Zhang et al., 2007)
Rab6	Golgi	no	23251	P{UAST-YFP.Rab6}CG10082[01]	(Zhang et al., 2007)
Rab7	Late endosomes	no	23270	P{UASp-YFP.Rab7}vig2[18]	(Zhang et al., 2007)
Rab7	Late endosomes	no	23641	P{UASp-YFP.Rab7}21	(Zhang et al., 2007)
Rab8		no	23272	P{UASp-YFP.Rab8}CG6175[09]	(Zhang et al., 2007)
Rab9		no	9784	P{UASp-YFP.Rab9}22	(Zhang et al., 2007)
Rab9Db		no	50774	P{UASp-YFP.CG9807}28	(Zhang et al., 2007)
Rab9E		no	9833	P{UASp-YFP.CG32673}35	(Zhang et al., 2007)
Rab9Fb		no	23276	P{UASp-YFP.Rab9Fb}how[Rab24]	(Zhang et al., 2007)
Rab10		no	9789	P{UASp-YFP.Rab10}21	(Zhang et al., 2007)
Rab11	Recycling endosomes	no	9790	P{UASp-YFP.Rab11}sra[32]	(Zhang et al., 2007)
Rab14		no	9794	P{UASp-YFP.Rab14}12	(Zhang et al., 2007)
Rab18		no	9796	P{UAST-YFP.Rab18}CG9775[01]	(Zhang et al., 2007)
Rab19		no	24150	P{UAST-YFP.Rab19}Hr39[02]	(Zhang et al., 2007)
Rab21		no	23242	P{UAST-YFP.Rab21}pog[04]	(Zhang et al., 2007)
Rab23		no	9802	P{UASp-YFP.Rab23}01	(Zhang et al., 2007)
Rab26		no	23244	P{UAST-YFP.Rab26}Lmpt[01]	(Zhang et al., 2007)
Rab27		no	24769	P{UASp-YFP.Rab27}14	(Zhang et al., 2007)
Rab30		no	9812	P{UASp-YFP.Rab30}Cdk4[10]	(Zhang et al., 2007)
Rab32		no	9815	P{UASp-YFP.ltd}RhoGDI[11]	(Zhang et al., 2007)
Rab35		no	9821	P{UASp-YFP.Rab35}15	(Zhang et al., 2007)
Rab39		no	9825	P{UASp-YFP.Rab39}13	(Zhang et al., 2007)
Rab40		no	9830	P{UAST-YFP.Rab40}Ptpmeg[03]	(Zhang et al., 2007)
RabX1		no	23274	P{UASp-YFP.RabX1}10	(Zhang et al., 2007)
RabX2		no	23275	P{UASp-YFP.RabX2}CG4936[08]	(Zhang et al., 2007)
RabX4		no	9851	P{UASp-YFP.RabX4}how[19]	(Zhang et al., 2007)
RabX5		no	9854	P{UASp-YFP.RabX5}eRF1[22b]	(Zhang et al., 2007)
RabX6		no	23278	P{UASp-YFP.RabX6}03	(Zhang et al., 2007)
Galt	Golgi	no	30902	UAS-Galt-GFP	

KDEL	Endoplasmic reticulum	no	9898	P{UAS-GFP.KDEL}11.1	
FYVE	PI(3)P-bearing endosomes	no	42712	P{UAS-GFP-myc-2xFYVE}2	
hLC3	Autophagosomes	no	8730	P{UASp-eGFP-huLC3}1	
Atg8a	Autophagosomes	no	52005	P{UAS-Atg8a.GFP}2	
Lamp1	Lysosomes	no		tub-LAMP1-GFP	(Akbar et al., 2009)
Lamp1	Lysosomes	no		UAS-LAMP1-GFP	(Pulipparacharuvil et al., 2005)
nSyb	Synaptic vesicles	no	6922	P{UAS-nSyb.eGFP}3	
Grasp65	Golgi	no	8508	P{UAS-Grasp65-GFP}3	
SKL	peroxisomes	no	28882	P{UAS-GFP.SKL}3	
gammaCOP	Golgi	no	29711	P{UASp-gammaCOP.EGFP}3	
mCD8	membrane and secretory vesicles	no	5130	P{w[+mC]=UAS-mCD8::GFP.L}LL6	
Chc	Clathrin coated vesicles	no		UAS-EGFP-Chc	(Burgess et al., 2011)
Clc	Clathrin coated vesicles	no	7101	P{w[+mC]=UAS-EGFP-Clc}3	
Sqh	Mysosin II	no	42234	P{GFP-sqh.RLC}40	
Cnn	Centrosomes	no	7255	P{UASp-GFP-Cnn1}26-1	
Snx3	Retromer	rare		UAS-Snx3-GFP	(Zhang et al., 2011)
Vps35	Retromer	some		UAS-Vps35-Myc	(Belenkaya et al., 2008)
Vps29	Retromer	rare		tub-vps29GFP	(Burgess et al., 2012)
Wls	Membrane/retromer	no		UAS-Wls-V5	(Belenkaya et al., 2008)
Ena	Adherens junctions	no	28798	w[*]; P{w[+mC]=Ubi-GFP.ena}3	
Cindr	Intercellular bridges, apical membrane	yes	50802	y[1] w[*]; P{w[+mC]=PTT-GA}cindr[CA06686]	
Scraps	Anillin	no	51348	P{w[+mC]=UASp-GFP-scra}3	
Septin2	Septin2	no	26257	P{w[+mC]=Sep2-GFP.SG}3	
Pav	Ring canals	no		ubi-pav-GFP	(McLean and Cooley, 2013)
Pod1	Actin/Microtubules	no	8800	w[*]; P{w[+mC]=UAS-pod1.GFPmyc}3/TM3, Sb[1]	
Klp61F	Mitotic spindles	no	35509	w[*]; P{w[+mC]=Ubi-Klp61F.GFP}3	

Akbar, M.A., Ray, S., and Kramer, H. (2009). The SM protein Car/Vps33A regulates SNARE-mediated trafficking to lysosomes and lysosome-related organelles. Molecular biology of the cell *20*, 1705-1714.

Belenkaya, T.Y., Wu, Y., Tang, X., Zhou, B., Cheng, L., Sharma, Y.V., Yan, D., Selva, E.M., and Lin, X. (2008). The retromer complex influences Wnt secretion by recycling wntless from endosomes to the trans-Golgi network. Developmental cell *14*, 120-131.

Burgess, J., Del Bel, L.M., Ma, C.I., Barylko, B., Polevoy, G., Rollins, J., Albanesi, J.P., Kramer, H., and Brill, J.A. (2012). Type II phosphatidylinositol 4-kinase regulates

trafficking of secretory granule proteins in Drosophila. Development *139*, 3040-3050.
Burgess, J., Jauregui, M., Tan, J., Rollins, J., Lallet, S., Leventis, P.A., Boulianne, G.L., Chang, H.C., Le Borgne, R., Kramer, H., *et al.* (2011). AP-1 and clathrin are essential for secretory granule biogenesis in Drosophila. Molecular biology of the cell *22*, 2094-2105.
McLean, P.F., and Cooley, L. (2013). Protein equilibration through somatic ring canals in Drosophila. Science *340*, 1445-1447.
Pulipparacharuvil, S., Akbar, M.A., Ray, S., Sevrioukov, E.A., Haberman, A.S., Rohrer, J., and Kramer, H. (2005). Drosophila Vps16A is required for trafficking to lysosomes and biogenesis of pigment granules. Journal of cell science *118*, 3663-3673.
Zhang, J., Schulze, K.L., Hiesinger, P.R., Suyama, K., Wang, S., Fish, M., Acar, M., Hoskins, R.A., Bellen, H.J., and Scott, M.P. (2007). Thirty-one flavors of Drosophila rab proteins. Genetics *176*, 1307-1322.
Zhang, P., Wu, Y., Belenkaya, T.Y., and Lin, X. (2011). SNX3 controls Wingless/Wnt secretion through regulating retromer-dependent recycling of Wntless. Cell research *21*, 1677-1690.
