# Supplementary material for: The Drosophila F-box protein Fbxl7 binds to the protocadherin Fat and regulates Dachs localization and Hippo signaling
Source: eLife. 2014 Aug 8;3:e03383. doi: 10.7554/eLife.03383 (PMC4144329; doi:10.7554/eLife.03383)
Supplement: Supplementary file 2. — Sequence of oligonucleotides used. DOI: http://dx.doi.org/10.7554/eLife.03383.020 [file elife03383s002.pdf]

Supplementary File 2 - Oligo Table

Oligo name	Sequence
Not1-Fbxl7-F	CGGCGGCGGCCGCTTCGCACAAGACTAGCAACCG
Xba1-Fbxl7-R	GCCGTCTAGATCAACAGAATCCCGGATTTG
C616Y-SDM-F	GTGTGTCCGACTACCTGAACATCAC
C616Y-SDM-R	GTGATGTTCAGGTAGTCGGACACAC
Not1-Fbxl7Δ1-F	CGGCGGCGGCCGCTTGGAACCGCAAAGGTCCC
Not1-Fbxl7Δ2-F	CGGCGGCGGCCGCTTCGCTGCGGGGCGAGCACT
Xba1-Fbxl7Δ3-R	GCGGTCTAGATTCAGGGCGGCGGTCCAATGGC
Not1-GFP-F	GGCGGAGGTGCGGCCGCTGTGAGCAAGGGCGAGGAG
Xba1-GFP-R	ACAAAGATCCTCTAGATCACTTGTACAGCTCGTCCA
Not1-SkpA-F	CGCGTCGCGGCCGCCACCATGCCCAGCATCAAGTTGC
Xba1-SkpA-R	GCCGTCTAGAGACTTCTCCTCGCACCACTCGT
Kpn1-Cul1-F	CTAGTGGTACCCACCATGAACCGCTCCGGCAAT
Not1-Cul1-R	GACGCGGCGGCCGCTGGCGAGATAACTATATGTGTCTTTG
Not1-Cul1DN-R	GACGCGGCGGCCGCTCACGACCATCACCTGGTTAAGA
Not1-FatICD-F	CGTCGCGGCCGCAAAATGGAGAGGCTACTGCTCC
Xba1-FatICD-R	GCGCGTCTAGATTACGTAGAATCGAGACCGAGGA
Fat61SDM-F	GCAGCCGCGCATTCTCATTTTGCACGACATTTCCGG
Fat61SDM-R	CCGGAAATGTCGTGCAAAATGAGAATGCGCGGCTGC
EcoRI-HA-Ub-F	CGCGTCGAATTCCAAAATGTACCCATACGATGTTCCAGATTAC GCTCAAATTTTCGTTAAGACCCTCACTG
Xba1-Ub-R	GCCGTCTAGACTAGCCACCACGCAGACGCAG
Not1-cindr-F	CGCGTCGCGGCCGCCACCATGGAAAACAACATCTGTGCA
Xba1-cindr-R	GCCGTCTAGAGAAACTTGCGTCACGCACTG
DRSC15513-F	TAATACGACTCACTATAGGGCGACCTTTGAGTTGAGAGG
DRSC15513-R	TAATACGACTCACTATAGGGGCAGTCGCTCGTTCCC
DRSC38270-F	TAATACGACTCACTATAGGGATCTGAACGCTCGAGGATGT
DRSC38270-R	TAATACGACTCACTATAGGGCCAAAAGTCCGTGTATGGCT
GFPdsRNA-F	TAATACGACTCACTATAGGGATGGTGAGCAAGGGCGAG
GFPdsRNA-R	TAATACGACTCACTATAGGGCTTGTACAGCTCGTCCATGC
